# Regulation of E-cadherin localization by microtubule targeting agents: rapid promotion of cortical E-cadherin through p130Cas/Src inhibition by eribulin

**DOI:** 10.18632/oncotarget.23798

**Published:** 2017-12-31

**Authors:** Nicholas F. Dybdal-Hargreaves, April L. Risinger, Susan L. Mooberry

**Affiliations:** ^1^ Department of Pharmacology, University of Texas Health Science Center at San Antonio, San Antonio, Texas, USA; ^2^ UT Health Cancer Center, University of Texas Health Science Center at San Antonio, San Antonio, Texas, USA

**Keywords:** microtubule targeting agents, eribulin, paclitaxel, E-cadherin, epithelial-to-mesenchymal transition

## Abstract

Microtubule targeting agents (MTAs) are some of the most effective anticancer drugs used to treat a wide variety of adult and pediatric cancers. Building evidence suggests that these drugs inhibit interphase signaling events and that this contributes to their anticancer actions. The effects of diverse MTAs were evaluated following a 2 hour incubation with clinically relevant concentrations to test the hypothesis that these drugs rapidly and differentially disrupt epithelial-to-mesenchymal transition (EMT)-related signaling. The MTAs rapidly promoted the cortical localization of internal pools of E-cadherin in HCC1937 breast cancer cells, with the most robust effects observed with the microtubule destabilizers eribulin and vinorelbine. Cortical E-cadherin localization was also promoted by the Src kinase inhibitor dasatinib or by siRNA-mediated depletion of the p130Cas scaffold. Mechanistic studies demonstrate that eribulin disrupts the interaction between p130Cas and Src, leading to decreased cortical Src phosphorylation that precedes the accumulation of cortical E-cadherin. These results suggest that microtubules can be actively co-opted by cancer cells to inhibit cortical E-cadherin localization, a hallmark of EMT, and provide a direct link between the initial disruption of the microtubule network and reversal of EMT phenotypes demonstrated by eribulin in long-term studies.

## INTRODUCTION

Microtubule targeting agents (MTAs) are highly effective anticancer drugs that remain a mainstay in the treatment of adult and pediatric cancers [1]. MTAs are classified as microtubule stabilizers or destabilizers based on their respective abilities to stimulate or inhibit the polymerization of tubulin and thereby increase or decrease the density of cellular microtubules leading to disruption of normal microtubule function. Microtubule stabilizers used clinically include the taxanes and the epothilone, ixabepilone, which all bind within the taxane site on microtubules [2]. Microtubule destabilizers with clinical anticancer activities include the vinca alkaloids and eribulin [3]. MTAs have significant utility in the treatment of breast cancer; however, even the structurally similar taxanes, paclitaxel and docetaxel, have some different clinical features and patients can respond to one of these drugs following failure on another [4, 5]. Divergent clinical responses to different MTAs was further highlighted during the development of eribulin, a MTA that provided a survival benefit as third-line therapy in patients with locally recurrent or metastatic breast cancer who had been treated previously with a taxane and an anthracycline [6]. These findings suggest that there are differences among MTAs that result in variations in patient responses. Identification of the mechanisms underlying these differences could lead to use of specific MTAs in patients with defined tumor characteristics to optimize the rational use of these drugs.

While all MTAs inhibit microtubule dynamics, there are significant differences in their effects on microtubule structure. Biochemical, X-ray crystallography, and, most recently, cryo-electron microscopy studies highlight the extensive structural heterogeneity of microtubules when they are perturbed by diverse MTAs [7-10]. Recently, near atomic resolution cryo-electron microscopy maps of microtubules stabilized by 3 chemically diverse microtubule stabilizers show that they initiate different effects on the microtubule lattice that affect lateral protofilament contacts [10]. These MTA-induced modifications in microtubule structure would be expected to differentially alter their function.

Microtubules serve as the tracks for transport of proteins, vesicles and organelles; consequently, it is not surprising that that disruption of microtubules interrupts cellular transport. In cancer cells, trafficking of p53, mRNA, the androgen receptor, and DNA repair proteins are altered by MTAs [11-15]. It is reasonable to assume that the differences in microtubule structure elicited by distinct MTAs could differentially influence the ability of microtubule binding proteins (MAPs), including dyneins and kinesins, to bind and transport cargo along microtubules. Indeed, different MTAs have distinct effects on mitochondrial and vesicular transport along axons [16, 17]. The unique ability of eribulin to bind only at the plus-ends of microtubules specifically affects the association of accessory microtubule plus-end tracking (TIP) proteins, including EB1 and EB3 [18]. Loss of these proteins at the plus-end of the microtubule prevents CLIP-170 binding, which is required for recruitment of dynein/dynactin motor proteins and loading of cargo for transport [19].

Microtubules also serve as cellular signaling hubs and the ability of MTAs to inhibit interphase signaling events likely contributes significantly to their anticancer actions [20, 21]. Paclitaxel disrupts the intracellular transport of K-Ras [22], which led to the finding that Ras inhibitors are particularly effective in combination with MTAs in Ras-driven cell lines and tumors [23]. Signal transduction is often coordinated by cellular scaffold proteins that spatially and temporally organize multiple signaling partners. Several scaffolds, including NEDD9 and IQGAP1, are associated with microtubules [24-26]. In breast cancer models the expression of the Cas family scaffold members NEDD9 (HEF1, Cas-L) or p130Cas (BCAR1) promotes phenotypes associated with epithelial-to-mesenchymal transition (EMT) [27] in a Src kinase-dependent manner [27-29]. Src has been shown in many contexts to promote EMT in part due to its ability to inhibit E-cadherin [30]. Importantly, the p130Cas-Src complex promotes the internalization and degradation of E-cadherin as a key-initiating event in EMT progression [31]. Microtubules coordinate E-cadherin-mediated cell adhesion [32] and the microtubule destabilizer eribulin has been shown to reverse EMT in triple negative breast cancer (TNBC) cell lines and a xenograft model [33], however the mechanistic link between acute microtubule disruption and E-cadherin localization has not been fully explored.

The downstream effects of MTAs on cellular signaling pathways have primarily been investigated several hours to days after drug treatment even though MTAs rapidly inhibit microtubule dynamics, within 3 seconds after drug addition in the case of eribulin [18]. Our goal is to identify the early effects of microtubule disruption by diverse MTAs on interphase oncogenic signaling pathways to test the hypothesis that MTAs rapidly and differentially disrupt microtubule-dependent signaling and trafficking events. We observed marked differences in the effects of MTAs on the redistribution of internal E-cadherin to the plasma membrane in HCC1937 TNBC cells within 2 hours of drug treatment. Further studies identified inhibition of the p130Cas-Src signaling complex as a component of the mechanism underlying E-cadherin redistribution and suggest that the EMT reversal observed after extended periods of eribulin treatment [33] could be initiated by disruption of the p130Cas signaling scaffold.

## RESULTS

### Identification of a short-term MTA treatment paradigm

**Figure 1:**
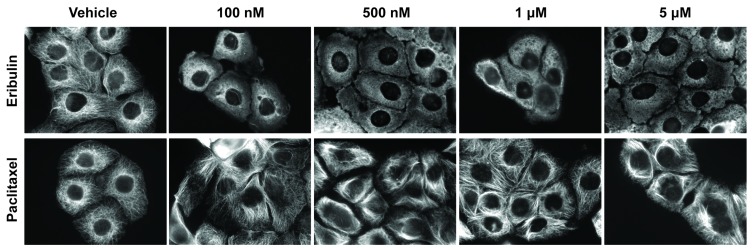
Concentration dependent effects of MTAs on interphase microtubules HCC1937 cells were treated for 2 hours with eribulin (top row) or paclitaxel (bottom row) at indicated concentrations. Cells were fixed with MeOH and microtubules visualized using indirect immunofluorescence with a β-tubulin antibody. Images are composed of non-deconvolved stacks.

**Figure 2:**
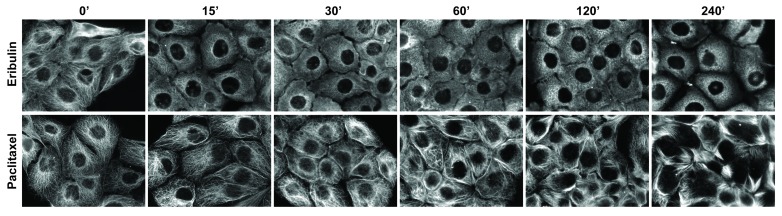
Time dependent effects of MTAs on interphase microtubules HCC1937 cells were treated with 100 nM eribulin (top row) or 1 μM paclitaxel (bottom row) for 0 - 240 minutes. The cells were fixed with MeOH and microtubules were visualized by indirect immunofluorescence using a β-tubulin antibody. Images are composed of non-deconvolved stacks.

To determine the early signaling and trafficking consequences of MTA-mediated microtubule disruption that occur rapidly, the temporal and concentration-dependent effects of 5 clinically approved MTAs used to treat breast cancer were evaluated. These drugs include 2 microtubule destabilizers: eribulin (Halaven) and vinorelbine (Navelbine), and 3 microtubule stabilizers: paclitaxel (Taxol), docetaxel (Taxotere), and ixabepilone (Ixempra). These MTAs represent 4 distinct chemical families and they were selected based on their differential effects on microtubule structure as well as their non-completely overlapping clinical activities. Initial evaluations of the effects of these MTAs in a panel of 4 TNBC cell lines (MDA-MB-468, HCC1937, MDA-MB-231, and BT-549) demonstrated that each caused profound microtubule disruption within 2 hours. Representative images for the time and concentration-dependent effects of eribulin and paclitaxel on microtubule structures in HCC1937 (Figures 1, 2) and in BT-549 cells (Supplementary Figures 1, 2) are presented. These results show that a 2 hour treatment with eribulin or paclitaxel causes a concentration-dependent disruption of interphase microtubules in both cell lines (Figure 1, Supplementary Figure 1). Near maximal microtubule disrupting effects were observed with 100 nM eribulin or 1 μM paclitaxel in both the HCC1937 and BT-549 cells within 2 hours.

Further evaluations of the temporal effects of 100 nM eribulin or 1 μM paclitaxel on interphase microtubules were conducted in both cell lines over the time course of 15 minutes - 4 hours (Figure 2, Supplementary Figure 2). Eribulin and paclitaxel exerted near maximal effects on interphase microtubules in both cell lines within 2 hours. Subsequent studies confirmed that a 2 hour treatment with 100 nM of the destabilizers (eribulin and vinorelbine) or 1 μM of the stabilizers (paclitaxel, ixabepilone, and docetaxel) elicited substantial and comparable disruption of the interphase microtubule network in multiple TNBC cell lines, including HCC1937 (Supplementary Figure 3 and data not shown). Therefore, treatment of TNBC cells with either 100 nM of a microtubule destabilizer or 1 μM of a microtubule stabilizer for 2 hours was selected as the experimental paradigm to evaluate the acute effects of MTAs on interphase signaling pathways. These concentrations are within the plasma concentrations measured in patients during pharmacokinetic studies of these drugs [34-38].

### Effects of eribulin and other MTAs on E-cadherin localization

Previous studies conducted by Yoshida et al., showed that a 7 day treatment of TNBC cells with eribulin reversed the EMT phenotype, including an increased expression of E-cadherin [33]. The events leading from initial microtubule disruption to these significant phenotypic changes that occur following multi-day treatments are not yet known. Since microtubules have been implicated in the cortical localization of E-cadherin [39], we hypothesized that the acute disruption of the interphase microtubule network by MTAs could alter E-cadherin localization independent of changes in protein expression.

**Figure 3:**
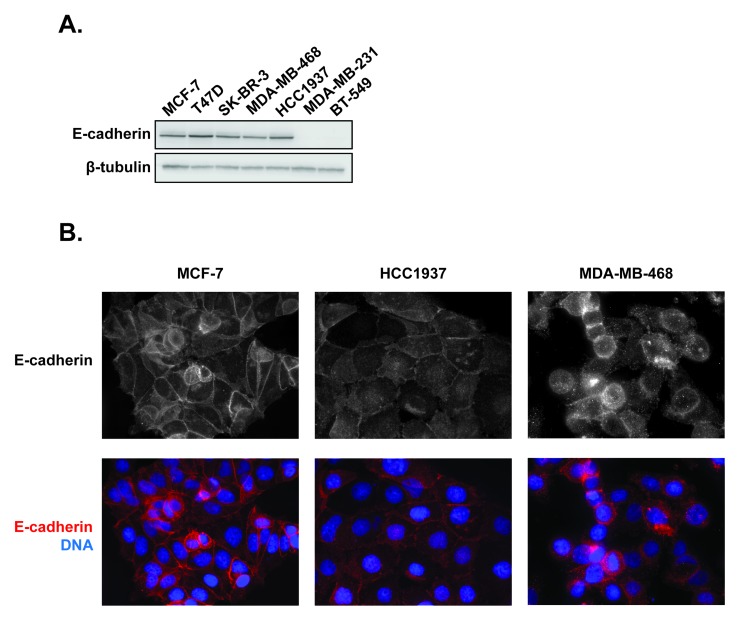
Expression and distribution of E-cadherin in a panel of 7 breast cancer cell lines **A.** Whole cell lysates of breast cancer cells were prepared and probed for E-cadherin expression by immunoblotting. **B.** The localization of E-cadherin was evaluated in estrogen receptor positive MCF-7 cells as well as HCC1937 and MDA-MB-468 triple negative breast cancer cells using indirect immunofluorescence. Images are composed of non-deconvolved stacks.

First, the basal expression of E-cadherin was determined in a panel of 7 breast cancer cells lines, including 4 TNBC cell lines, by immunoblotting. E-cadherin was detected in estrogen receptor positive (ER+) MCF-7 and T47D cells, HER2-expressing SK-BR-3 cells and in HCC1937 and MDA-MB-468 TNBC cells (Figure 3A). E-cadherin was not detected in MDA-MB-231 or BT-549 TNBC cells. The localization of E-cadherin was then evaluated in the epithelial-like MCF-7 cells as well as in the two TNBC cell lines that express E-cadherin (Figure 3B). As expected, the epithelial-like MCF-7 cells have robust cortical E-cadherin localization prevalent at contacts between adjacent cells, consistent with normal adherens junctions (Figure 3B). Although HCC1937 cells express similar levels of total E-cadherin by immunoblotting (Figure 3A), by immunofluorescence E-cadherin was distributed throughout the cytoplasm with little accumulation at cellular junctions (Figure 3B). These phenotypic results are consistent with the genomic studies that characterize HCC1937 as basal-like cells [40, 41], a hallmark of which is lack of cortical E-cadherin localization. [42]. In contrast, MDA-MB-468 cells presented a mixed phenotype with some cells exhibiting E-cadherin at membrane junctions while other cells did not (Figure 3B). Collectively, these data are consistent with previous findings that cell lines fall within distinct phenotypes along the EMT spectrum between epithelial and mesenchymal phenotypes [43]. The finding that HCC1937 cells express predominately internally localized E-cadherin places them in the middle of this spectrum between non-expressing and cortically localized E-cadherin and suggested that this cell line would be an ideal model to evaluate the effects of acute microtubule disruption on E-cadherin localization, independent of changes in expression.

**Figure 4:**
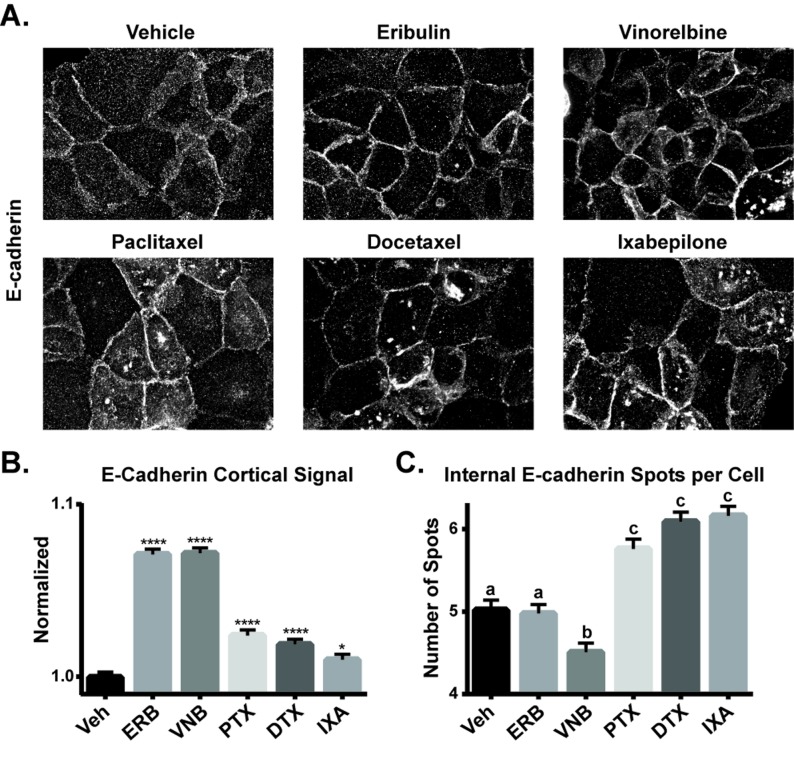
The effects of MTAs on E-cadherin localization **A.** E-cadherin localization was evaluated by indirect immunofluorescence in HCC1937 cells 2 hours after treatment with 100 nM eribulin or vinorelbine or 1 μM paclitaxel, docetaxel or ixabepilone. Images are composed of deconvolved stacks. **B.** The E-cadherin cortical signal was quantified in vehicle and drug-treated HCC1937 cells using high-content imaging. *N* = 2203-2608 ± SEM. ^*^p < 0.05, ^****^p < 0.0001. **C**. The internal E-cadherin spots were quantified in vehicle or drug-treated HCC1937 cells using high-content imaging. *N* = 1820-2347 ± SEM. Letters represent statistically identical groups.

The effects of the 2 hour MTA treatment on E-cadherin localization were then evaluated in HCC1937 and MCF-7 cells. In vehicle-treated HCC1937 cells, E-cadherin was localized throughout the cytoplasm with low staining at the periphery (Figure 4A). Treatment with eribulin or vinorelbine induced a dramatic redistribution of the E-cadherin to the cell cortex, particularly in areas of cell-cell contact (Figure 4A), similar to its localization in untreated, epithelial-like MCF-7 cells (Figure 3B). The microtubule stabilizers also promoted cortical E-cadherin accumulation, although less intense staining between cells was observed as compared to the effects of the destabilizers and, additionally, large internal E-cadherin inclusions were observed in many cells (Figure 4A).

High-content imaging was used to quantify the effects of the MTAs on E-cadherin localization. The cellular distribution of E-cadherin in the cell periphery was quantified as described in Supplementary Figure 4. Each MTA caused a significant increase in cortical E-cadherin localization, with the destabilizers causing the most robust increase (Figure 4B), consistent with the qualitative results shown in Figure 4A. A second analysis evaluated the number of large, internal E-cadherin spots among treatment groups (Supplementary Figure 4B). The stabilizers each caused a significant increase in the number of these E-cadherin inclusions compared to both vehicle and the destabilizers (Figure 4C). Studies showed that the E-cadherin inclusions initiated by paclitaxel treatment co-localized with the Golgi marker golgin97, but not with markers for other intracellular structures (Supplementary Figure 5A and data not shown). The MTA-induced E-cadherin localization changes occurred independent of changes in E-cadherin protein levels (Supplementary Figures 5B, 5C), confirming that MTAs rapidly alter the subcellular distribution of E-cadherin with differences noted among the drugs. In contrast, MTAs did not affect E-cadherin localization in the epithelial-like MCF-7 cells, where it remained at the cell periphery with either vehicle or MTA treatment (Supplementary Figure 6). A similar result was reported in the MDCK II renal epithelial cell line which exhibits E-cadherin at the cortex and this localization was also unaffected by MTAs [44].

**Figure 5:**
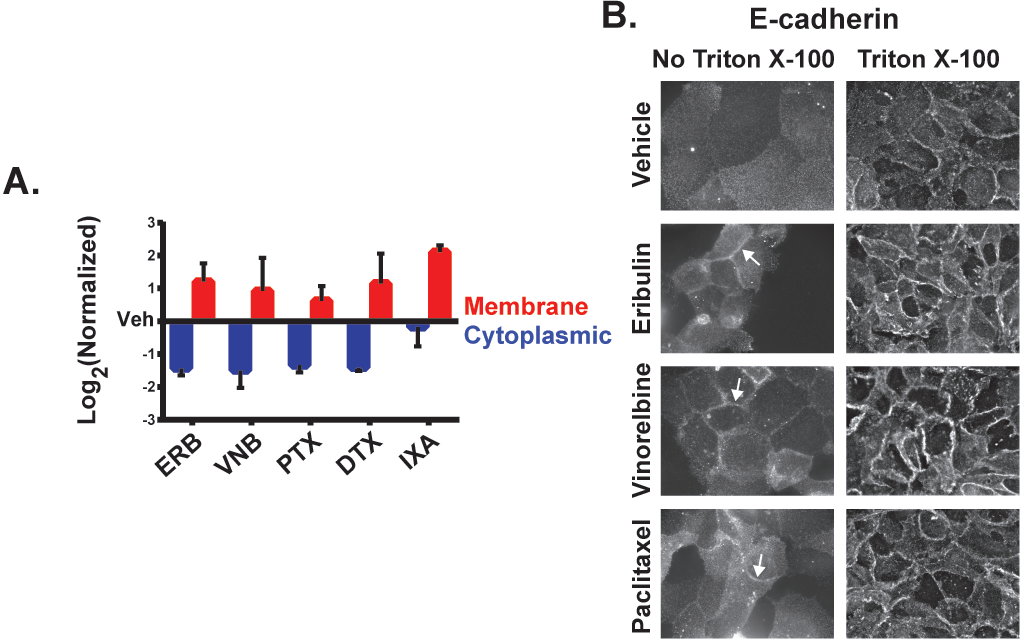
The effect of MTAs on the subcellular distribution of E-cadherin **A.** Membrane and cytoplasmic-enriched lysates of HCC1937 cells treated for 2 hours with vehicle or MTAs were prepared and analyzed by immunoblotting. Quantification of E-cadherin in the cytoplasmic and membrane-enriched fractions as compared to vehicle. *N* = 3 ± SEM. **B.** HCC1937 cells were prepared for indirect immunofluorescence with or without Triton X-100 permeabilization following 4% paraformaldehyde fixation. Arrows indicate E-cadherin ridges between cells in the absence of Triton X-100. Images are composed of non-deconvolved stacks.

To further evaluate the rapid changes in E-cadherin localization induced by MTAs, cytoplasmic and membrane-enriched fractions were generated and levels of E-cadherin in each fraction determined by immunoblotting. The separation of cytoplasmic and membrane-enriched fractions was confirmed by the distribution of the lipid raft protein flotillin in the membrane-enriched fraction and the cytoplasmic protein GAPDH in the cytoplasmic-enriched fraction (Supplementary Figure 7). A shift in E-cadherin from the cytoplasmic-enriched fraction to the membrane-enriched fraction as compared to vehicle-treated cells was observed with all MTAs (Figure 5A). These changes in E-cadherin distribution are consistent with the increased cortical E-cadherin observed in destabilizer-treated cells and the co-localization of E-cadherin with Golgi, which also sedimented with the membrane fraction, in stabilizer-treated cells.

To further confirm the localization of E-cadherin at the plasma membrane where it can form adherens junctions, E-cadherin immunofluorescence was performed in the absence of membrane permeabilization. Antibody impermeability in the absence of Triton X-100 detergent was demonstrated by a lack of intracellular β-tubulin staining under these conditions (Supplementary Figure 8). A lack of E-cadherin staining was also observed in vehicle-treated cells in the absence of detergent, indicating that E-cadherin was not readily accessible to the extracellular environment in these cells (Figure 5B). However, eribulin and vinorelbine promoted a pronounced accumulation of E-cadherin at cellular junctions in the absence of cell permeabilization within 2 hours, demonstrating a MTA-dependent change in the accessibility of E-cadherin to the extracellular environment (Figure 5B). Paclitaxel also promoted cortical E-cadherin accumulation, although to a lesser extent than eribulin or vinorelbine. These results collectively show that within 2 hours of treatment, MTAs can differentially induce E-cadherin localization to the cortical membrane in HCC1937 cells.

### p130Cas-Src signaling contributes to cytoplasmic E-cadherin localization

The ability of eribulin and other MTAs to rapidly induce phenotypic changes associated with EMT reversal in HCC1937 cells, independent of changes in total E-cadherin levels, suggests that these changes are mediated by disruption of microtubule-dependent signaling events that prevent localization of E-cadherin at the plasma membrane. Activated Src is a key regulator of E-cadherin localization, promoting loss of E-cadherin at adherens junctions [45, 46]. P130Cas is a cellular scaffold frequently overexpressed in breast cancers with poor prognosis [47] and it inhibits cortical E-cadherin stability in a Src-dependent manner [31].

**Figure 6:**
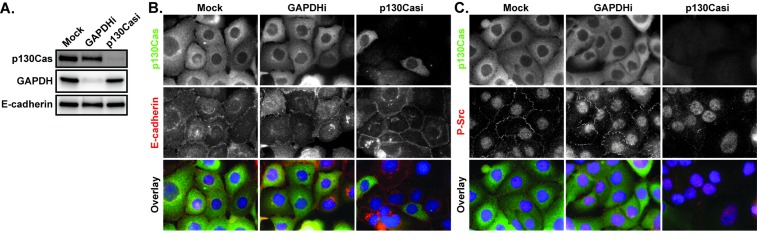
The effects of p130Cas knockdown on E-cadherin and P-Src **A.** HCC1937 cells were transfected with a mock control or siRNA directed against GAPDH or p130Cas and whole cell lysates evaluated by immunoblotting for p130Cas, GAPDH, and E-cadherin 72 hours later. **B.** Mock treated as well as p130Cas and GAPDH siRNA-treated cells (p130Casi and GAPDHi respectively) were probed for p130Cas (green), E-cadherin (red), and Hoescht (blue) by indirect immunofluorescence. **C.** Mock treated as well as p130Cas and GAPDH siRNA-treated cells were probed for p130Cas (green), P-Y419 Src (red), and Hoescht (blue) by indirect immunofluorescence. Images are composed of deconvolved stacks.

The role of p130Cas in regulating E-cadherin localization in HCC1937 cells was initially evaluated by siRNA-mediated p130Cas knockdown. Immunoblots confirmed siRNA-mediated knockdown of p130Cas levels in total cell lysates (Figure 6A). Immunofluorescence images showed that p130Cas was localized through the cytoplasm in mock and control GAPDH siRNA-treated HCC1937 cells and that siRNA-mediated p130Cas knockdown reduced p130Cas levels in the majority of cells (Figure 6B). As expected, E-cadherin was localized predominantly internally in mock or GAPDH siRNA transfected cells. However, depletion of p130Cas caused E-cadherin accumulation at the cortex (Figure 6B) with no associated change in total E-cadherin levels (Figure 6A). The localization of E-cadherin at the cell cortex in the absence of p130Cas is consistent with the role of this scaffold protein in promoting Src-mediated E-cadherin internalization [31]. Indeed, active P-Src (Y419 autophosphorylation) was localized to the cell periphery in mock and GAPDH siRNA transfected cells, but lost from the periphery in p130Cas-depleted cells (Figure 6C). These results are consistent with p130Cas playing a role in both cortical Src activation and the internal localization of E-cadherin in HCC1937 cells.

### Inhibition of Src recapitulates the effects of eribulin on E-cadherin localization

**Figure 7:**
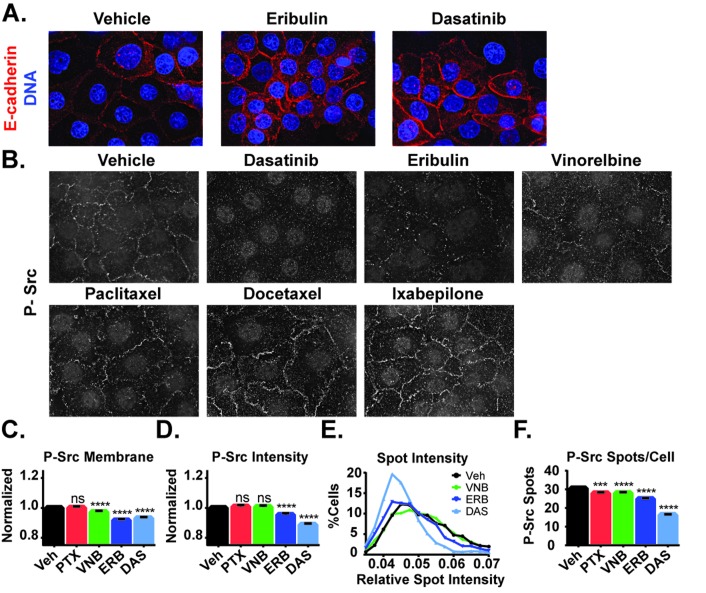
Effects of MTAs and the Src family kinase inhibitor dasatinib on E-cadherin and phospho-Y419 Src localization **A.** E-cadherin localization (red) was evaluated in HCC1937 cells treated for 2 hours with vehicle, 100 nM eribulin, or 25 nM dasatinib. **B.** P-Y419 Src localization was visualized in HCC1937 cells treated with 25 nM dasatinib, 100 nM eribulin or vinorelbine, or 1 μM paclitaxel, docetaxel, or ixabepilone by indirect immunofluorescence. Images are composed of deconvolved stacks. C.-F. The intensity and distribution of P-Y419 Src in vehicle and drug-treated HCC1937 cells were quantified using high-content imaging. **C**. Signals from the texture-based analysis of P-Src at the cell cortex were plotted and compared. **D**. The intensity of cytoplasmic P-Src spots in each of the treatment groups was compared. **E**.The distribution of the percentage of cells with a mean spot intensity was plotted as a histogram for vehicle (black), vinorelbine (green), eribulin (dark blue), and dasatinib (light blue) treatment conditions. **F**. The number of cytoplasmic P-Src spots per cell was plotted and compared. *N* = 1846-2276 ± 95% CI. ^***^p < 0.001, ^****^p < 0.0001.

To further explore the role of Src in the localization of E-cadherin after microtubule disruption, the effect of the Src family inhibitor dasatinib on E-cadherin localization was evaluated. A 2 hour treatment of HCC1937 cells with 25 nM dasatinib initiated a phenotype strikingly similar to that of eribulin with regard to the extensive cortical E-cadherin relocalization (Figure 7A). We therefore hypothesized that the MTA-induced redistribution of E-cadherin could be a result of alterations in Src localization or activity. The effects of MTAs on the levels and localization of P-Src were therefore evaluated and compared to dasatinib. Eribulin caused extensive reduction of P-Src at the cell periphery with only a few remnant P-Src foci in the regions between cells, similar to the effect of dasatinib (Figure 7B). Vinorelbine also reduced P-Src localization at the cellular cortex, albeit to a lesser extent than eribulin, while the microtubule stabilizers did not appear to have a large impact on P-Src at the cortex (Figure 7B).

The effects of the MTAs on cortical P-Src localization were further evaluated by high- content imaging. Eribulin and vinorelbine caused statistically significant inhibition of P-Src localization to the plasma membrane and the effect of eribulin was comparable to dasatinib (Figure 7C). Eribulin and dasatinib also significantly decreased the total cellular P-Src spot intensity, but vinorelbine did not (Figure 7D). Paclitaxel did not affect either P-Src localization to the plasma membrane or P-Src spot intensity. The differences among dasatinib, eribulin, and vinorelbine were further highlighted by comparing the distribution of P-Src spot intensity across the cell populations (Figure 7E). Dasatinib and eribulin each reduced the proportion of cells with intense P-Src staining as evidenced by the leftward shift in the peak and loss of cells with higher relative spot intensity as compared to vehicle or vinorelbine. When the total number of P-Src spots in a cell were quantified independent of their intensity, eribulin, vinorelbine, and paclitaxel all initiated a similar decrease in this measure, which was less than dasatinib (Figure 7F). Despite not affecting P-Src intensity or membrane localization, the finding that paclitaxel caused a significant decrease in the number of P-Src spots per cell suggests that microtubule stabilizers have a subtler effect on Src activity than the destabilizers. Together, these results indicate that microtubule disruption by diverse MTAs can differentially inhibit the cortical localization of activated Src, which could contribute to MTA-induced cortical E-cadherin localization.

### Eribulin inhibits the ability of p130Cas to colocalize and interact with Src

Eribulin caused the most robust inhibition of cortical P-Src among the MTAs evaluated (Figure 7), prompting a primary focus on this MTA in further mechanistic evaluations. The finding that either inhibition of Src signaling or eribulin treatment initiated the rapid localization of E-cadherin to the cortical membrane (Figures 6, 7) led to the hypothesis that eribulin could promote cortical E-cadherin localization by disrupting the interaction between P-Src and its scaffold, p130Cas. The ability of microtubule disruption to impact Cas-mediated scaffolding is consistent with the known functions of Cas scaffolds in cytoskeletal organization and, in some cases, their interactions with microtubules [24, 25, 48].

**Figure 8:**
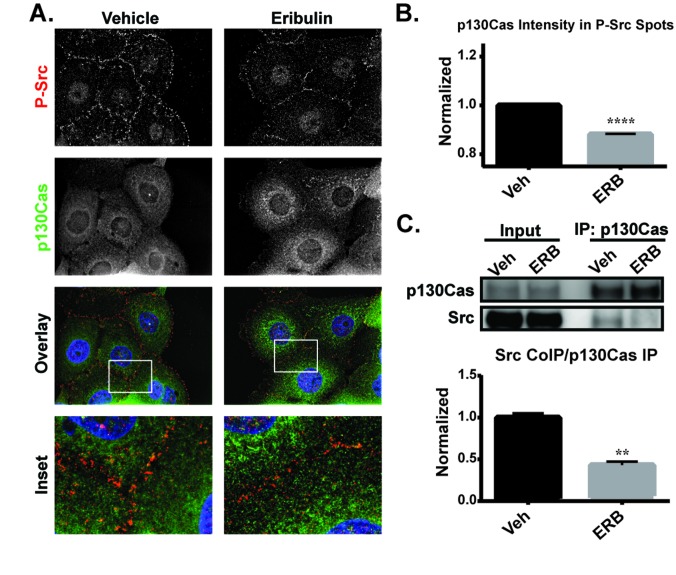
The effects of eribulin on the localization of P-Src and interaction of p130Cas with Src **A.** P-Y419 Src (red), p130Cas (green) and Hoechst (blue) were visualized by indirect immunofluorescence in HCC1937 cells treated for 2 hours with vehicle or 100 nM eribulin. Images are composed of deconvolved stacks. **B.** Quantification of the p130Cas signal within P-Src spots using high-content imaging. *N* = 7547-8931. **C.** Co-immunoprecipitation of Src with p130Cas from eribulin or vehicle-treated HCC1937 lysates. Src levels were quantified relative to p130Cas levels and quantified. *N* = 3 ± SEM. ^**^p < 0.01, ^****^p < 0.0001.

In vehicle-treated HCC1937 cells, P-Src was localized predominantly at the cell cortex and p130Cas was distributed throughout the cytoplasm with some cortically localized puncta (Figure 8A). The immunofluorescence images suggested the possibility that eribulin caused a reduction in co-localization of these proteins at the cell surface (Figure 8A, see inset). High-content imaging was used to address whether eribulin inhibited the co-localization of P-Src and p130Cas. First, the P-Src spots were identified and then the intensity of p130Cas localized within these defined regions was determined. The results show that eribulin caused a statistically significant decrease in the intensity of p130Cas within P-Src spots (Figure 8B). This loss of co-localization suggests that eribulin disrupts the p130Cas-Src interaction. To directly test this, p130Cas was immunoprecipitated from both vehicle and eribulin-treated cells and the extent of co-immunoprecipitation of Src evaluated. A 2 hour eribulin treatment was sufficient to significantly reduce the amount of Src that co-immunoprecipitated with p130Cas as compared to vehicle-treated cells (Figure 8C, Supplementary Figure 9). Collectively, these data demonstrate that eribulin-mediated microtubule depolymerization disrupts the p130Cas-Src interaction, thus altering p130Cas-Src signaling and leading to a reduction in active Src at the cell cortex.

### Loss of cortical P-Src precedes localization change in E-cadherin

**Figure 9:**
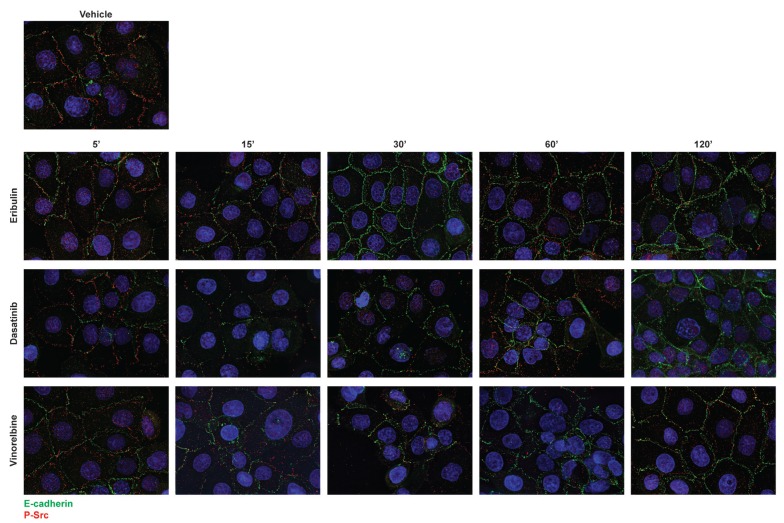
Kinetics of dasatinib and microtubule destabilizers on E-cadherin and P-Src localization HCC1937 cells were treated with vehicle, 25 nM dasatinib or 100 nM eribulin or vinorelbine for 5 - 120 minutes. Cells were then probed for E-cadherin (green), P-Y419 Src (red), and Hoescht (blue) by indirect immunofluorescence. Images are composed of deconvolved stacks.

Since microtubule disruption by eribulin causes E-cadherin cortical localization that is associated with a reduction in phosphorylated Src, we next evaluated whether the eribulin-mediated loss of cortical P-Src preceded E-cadherin localization to the cortex. The timing of these events in dasatinib, eribulin, and vinorelbine-treated cells was evaluated (Figure 9, Supplementary Figures 10, 11). Within 15 minutes, a near maximal loss of cortical P-Src was observed with dasatinib, eribulin, and vinorelbine treatment (Figure 9, Supplementary Figure 10). In contrast, E-cadherin localization 15 minutes after drug-treatment was not different from vehicle-treated cells, but was enhanced at the cortical membrane within 30 minutes - 2 hours (Figure 9, Supplementary Figure 11). These results indicate that inhibition of peripheral Src precedes E-cadherin localization to the cortex and strongly supports the assertion that disruption of Src signaling by the microtubule destabilizers contributes to their ability to rapidly promote an epithelial-like distribution of E-cadherin.

### Eribulin and dasatinib are more effective in combination

**Figure 10:**
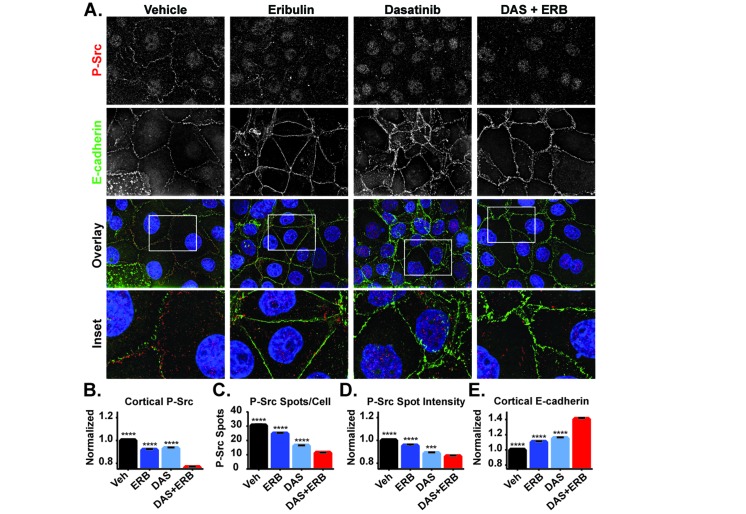
The effects of combined eribulin and dasatinib treatment on P-Src and E-cadherin localization **A.** The localization of P-Y419 Src (red) and E-cadherin (green) were evaluated by indirect immunofluorescence in HCC1937 cells treated for 2 hours with vehicle, 100 nM eribulin, 25 nM dasatinib, or the combination of both drugs. Images are composed of deconvolved stacks. B-E Quantification of P-Src and E-cadherin signals in vehicle and eribulin-treated cells using high-content imaging. **B.** P-Src at the cell periphery was analyzed using a texture-based analysis. **C.** The number of P-Src spots in the cytoplasm was quantified. **D.** The background-corrected average intensity of P-Src spots was quantified. **E.** The cortical E-cadherin texture-based signal was quantified. A Kruskal-Wallis analysis with a post hoc test was used in each analysis to compare vehicle and individual drug treatments to the combination of eribulin and dasatinib. *N* = 1846-2276 ± 95% CI. ^***^p < 0.001, ^****^p < 0.0001.

**Figure 11:**
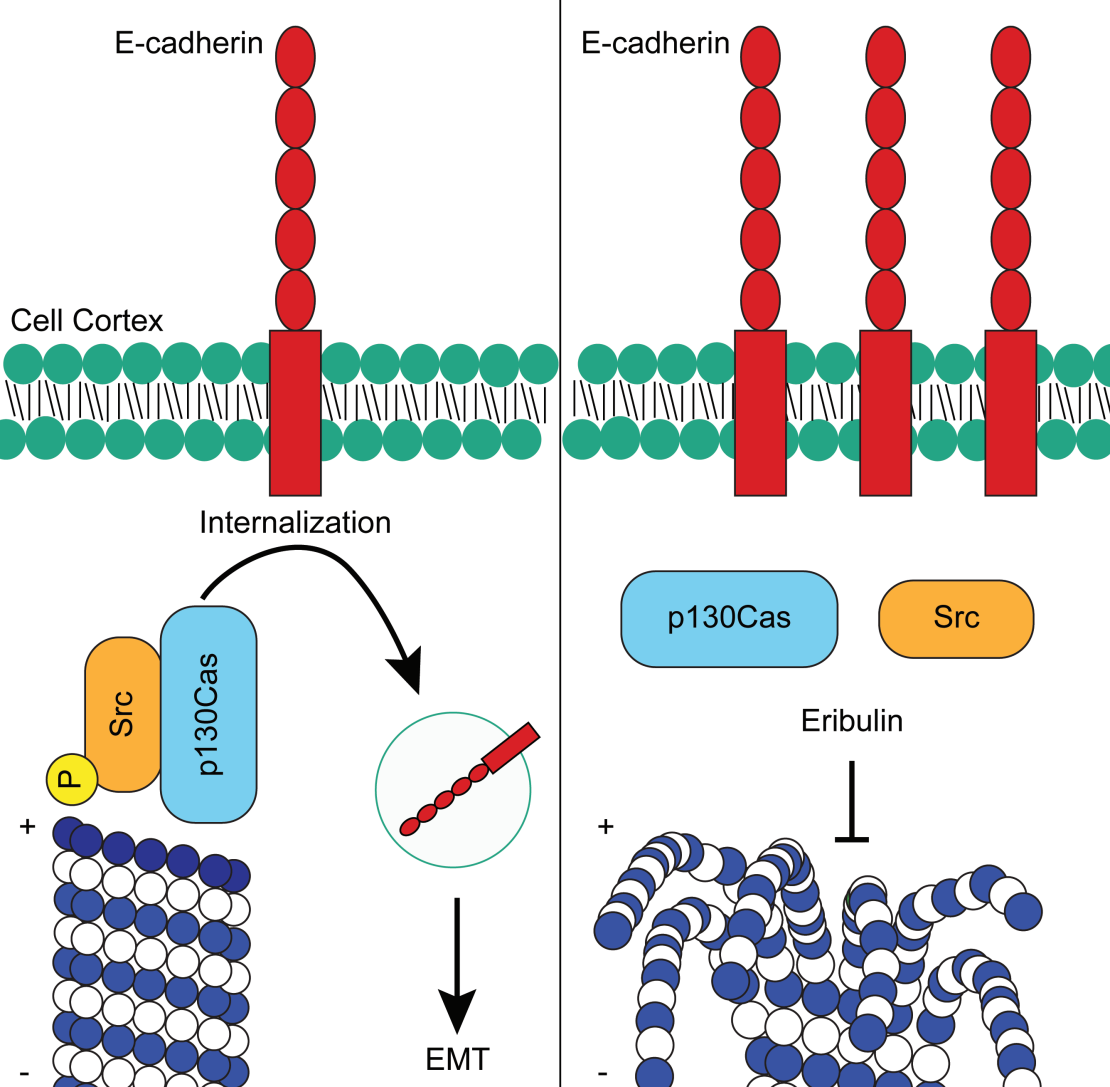
Proposed mechanism of how eribulin promotes cortical localization of E-cadherin in HCC1937 cells In untreated cells, activated P-Src localized with the p130Cas scaffold induces the internalization of E-cadherin from the cortex. Following depolymerization of the microtubule at the plus-end by eribulin, Src rapidly dissociates from p130Cas and becomes dephosphorylated. In the absence of active Src, E-cadherin is no longer internalized and accumulates at the cortex where it promotes EMT reversal.

Eribulin and dasatinib initiate cortical localization of E-cadherin in HCC1937 cells through inhibition of Src signaling, but do so through distinct mechanisms. Dasatinib directly inhibits Src tyrosine kinase activity while eribulin disrupts Src signaling by inhibiting its interaction with p130Cas (Figure 8). These distinct mechanisms of Src inhibition suggest that the combination of eribulin and dasatinib could be more effective than either drug alone at promoting epithelial E-cadherin localization, a hallmark of EMT reversal. The combination of eribulin and dasatinib was evaluated and found to be highly effective in causing loss of cortical P-Src and promotion of cortical E-cadherin localization (Figure 10A). Quantitative high-content imaging confirmed that the combination of eribulin and dasatinib caused a statistically significant inhibition of the P-Src membrane signal, the number of P-Src spots per cell, and relative intensity of those spots as compared to vehicle or either drug alone (Figure 10B-10D). The localization of E-cadherin at the cortex was also enhanced in the presence of combinations of dasatinib and eribulin as compared to either treatment alone (Figure 10E), consistent with their different mechanisms of inhibiting cortical Src. Cumulatively, our results show that eribulin-mediated microtubule depolymerization inhibits Src signaling by disrupting its interaction with the p130Cas scaffold leading to loss of the oncogenic, EMT-associated, signal that prevents cortical localization of E-cadherin. This proposed mechanism is summarized in Figure 11.

## DISCUSSION

These studies were designed to evaluate the early effects of microtubule disruption on oncogenic signaling pathways that drive EMT, which is associated with a poor prognosis in metastatic breast cancer [49]. Accumulating evidence demonstrates that the anticancer efficacy of MTAs is not due solely to their antimitotic effects, but also involves their numerous effects on cellular trafficking and signaling [20, 21]. In contrast to the majority of studies that looked at the effects of MTAs 18-24 hours, or even days, after drug addition, we investigated the early consequences of microtubule disruption to identify initiating signaling events elicited by MTAs while avoiding the confounding consequences of mitotic accumulation. While this approach differs from many, a nearly identical 2 hour treatment paradigm with MTAs at similar concentrations was reported to induce JNK/SAPK signaling [50]. Our studies show that a 2 hour treatment with MTAs at clinically relevant concentrations [34-38] was sufficient to elicit extensive microtubule disruption that inhibited the Src-p130Cas interaction leading to cortical redistribution of E-cadherin. While it is likely that these changes in E-cadherin localization could lead to functional changes to cell migration and invasion, these effects cannot be separated from the well-documented ability of MTAs to directly inhibit cell motility [51, 52].

Yoshida and colleagues previously demonstrated that a 7 day treatment of mesenchymal TNBC cells with eribulin altered the morphology of surviving cells to a more epithelial phenotype and increased the expression of multiple epithelial markers, including E-cadherin with a concomitant decrease in mesenchymal markers [33]. A similar result was also reported with a 2-day treatment of bladder cancer cells with vinflunine [53]. In addition to the ability of microtubule destabilizers to induce the expression of E-cadherin after multi-day treatments, our findings demonstrate that MTAs can rapidly promote the cortical localization of internal pools of E-cadherin, which is required for proper coordination of cell-cell adhesion. This finding is consistent with results from the Gumbiner laboratory showing that microtubules inhibit the adhesive function of E-cadherin, mediated by activated p120 catenin, in Colo 205 colorectal carcinoma cells [32]. Extensive preclinical studies have shown that cortically localized E-cadherin can inhibit EMT progression. Ligated E-cadherin has been shown to transmit contact inhibition and in this way suppress Wnt and EGFR signaling, both key EMT signaling pathways [54]. In addition, E-cadherin loss is sufficient to induce EMT and permit cells to gain metastatic capabilities [55]. Furthermore, loss of E-cadherin at the cortical membrane is a major predictor of poor prognosis in breast cancer and therapies that promote E-cadherin to cellular junctions are actively being sought [56]. Interestingly, the HCC1937 line was derived from a stage 2B, grade 3 tumor that had not metastasized [57]. This suggests that HCC1937 cells may represent an intermediate phenotype of an aggressive, but non-metastatic, tumor that has lost proper E-cadherin localization but not expression. Understanding the multiple mechanisms by which eribulin and other MTAs promote cortical E-cadherin localization could lead to their targeted use in patients with low E-cadherin expression or those whose E-cadherin is expressed but localized internally, as is the case for the HCC1937 model used in this study. However, it was recently shown that the adhesive activity of E-cadherin, not just its expression, is ultimately the most critical for inhibition of metastasis [58].

Our results showing that microtubule disruption rapidly alters the distribution of E-cadherin in HCC1937 cells lends additional evidence that microtubules can be actively co-opted by cancer cells to promote EMT-associated phenotypes, including internalization of E-cadherin. The finding that microtubule disruption by eribulin, p130Cas knockdown, and direct Src inhibition with dasatinib each increased the cortical localization of E-cadherin suggests that a common pathway was disrupted. The eribulin-mediated loss of P-Src at the cortex and disruption of both the co-localization and co-immunoprecipitation of p130Cas with Src provides further support that eribulin disrupts the p130Cas-Src signaling axis and that this contributes to its ability to induce rapid cortical localization of E-cadherin. The ability of eribulin to disrupt the interactions between Src and p130Cas to promote cortical E-cadherin localization within just 2 hours of drug treatment is consistent with the fact that Src is actively transported along microtubules [59] and that p130Cas, which scaffolds Src to facilitate its downstream signaling [60], has previously been shown to promote E-cadherin removal from the plasma membrane [31].

Each of the 5 MTAs evaluated had effects on E-cadherin and P-Src localization, however significant differences were noted among them. The MTAs all enhanced membrane-associated E-cadherin, yet cortical localization was more prominent in cells treated with the destabilizers eribulin and vinorelbine, while the stabilizers, paclitaxel, docetaxel and ixabepilone, also enhanced the accumulation of E-cadherin in large internal punctate structures that colocalized with the Golgi. Subtle differences were noted even among the 3 stabilizers with ixabepilone having effects that differed most from those initiated by the destabilizers. The differences between paclitaxel and eribulin in their promotion of cortical E-cadherin and P-Src localization might be associated with the ability of eribulin to reverse EMT phenotypes [33] and the EMT pathway response associated with sensitivity to eribulin but not paclitaxel in breast cancer cell lines [61]. Consistent with our findings, the Gumbiner laboratory found that nocodazole, but not paclitaxel-mediated microtubule disruption, stimulated E-cadherin adhesion in Colo 205 cells, an effect mediated by dephosphorylation of p120 catenin [32]. Interestingly, Src has been shown to phosphorylate p120 catenin, suggesting the potential of a common pathway [58, 62].

It is noteworthy that eribulin was the most effective MTA in our panel at inhibiting p130Cas-Src signaling, a pathway implicated in EMT, since eribulin provides a survival advantage in breast cancer patients, including those with metastatic disease, which is often characterized by EMT [63]. Additionally, the finding that eribulin, a drug that specifically binds to the plus-end of microtubules and displaces plus-end binding proteins [64], had the most profound effect on E-cadherin localization in our panel of 5 MTAs is consistent with the demonstration that the plus-end binding protein CLIP-170 also promotes loss of cortical E-cadherin [65]. Although we cannot exclude the possibility that microtubule disruption by eribulin also impacts E-cadherin localization through additional Src-independent mechanisms, such as CLIP-170 displacement, the ability of eribulin to inhibit Src and promote E-cadherin localization to the cortical membrane in the same time frame as dasatinib suggests that the eribulin-mediated inhibition of p130Cas-Src complex contributes to the relocalization of E-cadherin to the cortex.

Our results demonstrating rapid cortical accumulation of E-cadherin following MTA treatment, with differences noted among drugs, highlights the ability of these drugs to rapidly inhibit cellular signaling and suggests the potential for future studies to define the selective effects of different MTAs on diverse signaling pathways. These proposed studies could begin to define whether specific MTAs could be used as more targeted therapies in different molecular contexts [66]. It is well established that patients can show different responses to specific MTAs, and failure on one MTA does not preclude sensitivity to another. The molecular underpinnings of these differences are only now being identified and data suggest they could be due to differences in the effects of these drugs on interphase signaling [11, 13, 59, 67, 68]. Although future studies will be necessary to define drug combinations that will be optimal in specific molecular contexts, our results expand the building evidence that MTAs disrupt oncogenic signaling. The further elucidation of the mechanistic actions of MTAs as targeted disruptors of oncogenic signaling will be critical to improving and personalizing the use of these highly effective anticancer drugs.

## MATERIALS AND METHODS

### Cell lines and reagents

BT-549 cells were obtained from the Lombardi Cancer Center at George Washington University, initially authenticated in April 2014 by STR-based profiling (Promega), and maintained in RPMI. All other cell lines were obtained directly from ATCC and authenticated most recently by Genetica in August 2017. Cells were grown in the following media: MCF-7, MDA-MB-231 and SK-BR-3 in IMEM; BT-549, HCC1937, MDA-MB-468 and T-47D in RPMI. All media contained 10% FBS with 0.25-0.5% gentamycin and cells were grown in a humidified 37°C incubator with 5% CO_2_. Cell stocks were stored in liquid nitrogen and all experiments performed on cells within 6 months of retrieval. Paclitaxel and docetaxel (Sigma Aldrich), ixabepilone (LC Labs), vinorelbine (AdooQ Bioscience), dasatinib (Selleckchem), and eribulin (Eisai Inc.) were dissolved in DMSO and stored at -20°C. Antibody information is provided in Supplemental Table 1.

### Indirect immunofluorescence

Cells plated on coverslips were treated with MTAs, fixed and permeabilized with ice-cold methanol (Figure 1 and 2 and Supplementary Figures 1 and 2) or fixed with 4% paraformaldehyde and permeabilized with 0.5% Triton X-100 (with the exception of experiments evaluating cell surface proteins (Figure 5B, Supplementary Figure 8 second row), which were detected using a 5 minute fixation without detergent in a manner similar to [44]), followed by immunostaining. A Nikon Eclipse 80i fluorescence microscope with NIS elements was used for image acquisition. All figures show stacked images; images in Figures 1, 2, 3, and 5 are not deconvolved whereas images in Figures 4, 6, 7, 8, 9, 10 are deconvolved using Nikon deconvolution software. For high-content imaging, a process that is defined as the automated measure of many subcellular parameters in parallel, cells were plated in 96-well image view plates (ThermoNunc) and imaged in a single focal plane using an Operetta® (PerkinElmer). A 20x LWD objective was used for acquisition and data were evaluated using Columbus™ (PerkinElmer). Nuclei were identified using Hoechst 33342 stain and cytosol defined by β-tubulin immunofluorescence. Spots or textures of cytosolic E-cadherin, P-Src and p130Cas were defined [69, 70]. Detailed descriptions of these analyses are in Supplementary Figure 4.

### Immunoblotting

Cells treated for 2 hours with MTAs were lysed in cell extraction buffer (ThermoFisher) with protease and phosphatase inhibitors. Equal amounts of protein were resolved by PAGE and evaluated by immunoblotting as previously described [71]. Membrane and cytosolic fractions were enriched using established methods [72]. Briefly, cells were homogenized using a Potter-Elvehjem homogenizer in a hypotonic homogenization buffer containing protease and phosphatase inhibitors, large debris was pelleted and then resulting supernatants were centrifuged at 16,000 g for 30 min. The final pellet was resuspended in homogenization buffer containing 1% Triton and designated as the membrane extract while the supernatant was designated the cytosolic extract.

### Co-immunoprecipitation

Cells treated for 2 hours with eribulin or vehicle were lysed in a CHAPS buffer containing protease and phosphatase inhibitors. Equivalent amounts of protein were precleared with protein G beads (GE Healthcare), immunocomplexes containing p130Cas-IgG or control-IgG (BD Biosciences) were formed for 2 hours and then protein G beads used to precipitate the immunocomplexes. Beads were washed and prepared for immunoblotting.

### siRNA transfection

HCC1937 cells were transfected using Lipofectamine RNAiMAX as recommended by the manufacturer (ThermoFisher). siRNAs against GAPDH (AM4605 ThermoFischer) and p130Cas (SASI_Hs01_00184840 and SASI_Hs02_00345830 (Sigma Aldrich) were used.

### Statistics

Distributions of data were tested for normality and then an appropriate multivariate test (ANOVA or Kruskal-Wallis) was used (Prism 6, GraphPad). Post hoc multiple comparison testing was performed comparing each treatment to the vehicle control, the combination of eribulin and dasatinib, or all pair-wise comparisons depending on the experiment (*P* < 0.05). Unless otherwise noted, experiments were performed in triplicate.

## SUPPLEMENTARY MATERIALS FIGURES AND TABLE


