# Correction: Regulation of E-cadherin localization by microtubule targeting agents: rapid promotion of cortical E-cadherin through p130CAS/Src inhibition by eribulin

**DOI:** 10.18632/oncotarget.27100

**Published:** 2019-07-23

**Authors:** Nicholas F. Dybdal-Hargreaves, April L. Risinger, Susan L. Mooberry

**Affiliations:** ^1^ Department of Pharmacology, University of Texas Health Science Center at San Antonio, San Antonio, Texas, USA; ^2^ UT Health Cancer Center, University of Texas Health Science Center at San Antonio, San Antonio, Texas, USA

**This article has been corrected:** The authors recently became aware that the antibody used for Figure 5B was inappropriate to address the ability of E-cadherin to be accessible from the extracellular space in the absence of Triton X permeabilization. The E-cadherin antibody, DECMA-1 from EMD Millipore (Cat. # MABT26) which has the ability to block E-cadherin-associated cell adhesion, is appropriate to address this question. Multiple experiments were conducted with the DECMA-1 antibody in the presence and absence of Triton X permeabilization. New pictures were taken, and a revised Figure 5B using these new images is shown below. A revised Supplementary Table 1 with a list of the antibodies used for each figure is also shown. The authors declare that these corrections do not change the results or conclusions of this paper.

 Original article: Oncotarget. 2018; 9:5545–5561. 5545-5561 . https://doi.org/10.18632/oncotarget.23798

**Figure 5:**
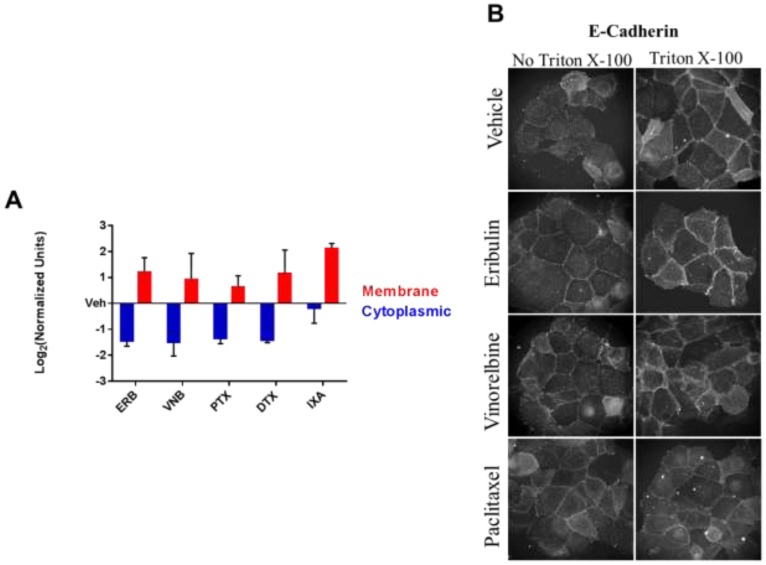
The effect of MTAs on the subcellular distribution of E-cadherin. **A.** Membrane and cytoplasmic-enriched lysates of HCC1937 cells treated for 2 hours with vehicle or MTAs were prepared and analyzed by immunoblotting. Quantification of E-cadherin in the cytoplasmic and membrane-enriched fractions as compared to vehicle. *N* = 3 ± SEM. **B.** HCC1937 cells were prepared for indirect immunofluorescence with or without Triton X-100 permeabilization following 4% paraformaldehyde fixation. Arrows indicate E-cadherin ridges between cells in the absence of Triton X-100. Images are composed of non-deconvolved stacks.

**Supplementary Table 1:** Antibody Vendors and Applications

Host-Target	Vendor Information	Host	Figures Used	Application-Dilutions*
E-cadherin E-cadherin	3195S, Cell Signaling DECMA-1, EMD Millipore	Rabbit Rat	3, 4, 6, 7, S5, S6, S7, S8 5	IF- 1:400 W- 1:1000 IF-1:400
GAPDH	5174S, Cell Signaling	Rabbit	6, S5, S7	W- 1:1000
β-tubulin	ab6046, Abcam	Rabbit	3	W- 1:1000
β-tubulin	T-4026, Sigma Aldrich	Mouse	1, 2, S1, S2, S3, S4, S8	IF- 1:400
E-cadherin	5296S, Cell Signaling	Mouse	9, 10, S11	IF- 1:400
Flotillin	BD Bioscience	Mouse	S7	W- 1:1000
Golgin97	A21270, Invitrogen	Mouse	S5	IF-1:200
p130Cas	ab31831, Abcam	Mouse	6, 8, S9	IF- 1:400 W- 1:1000 IP- 2 µg
P-Y418 Src	ab4816, Abcam	Rabbit	6, 7, 8, 9, 10, S4, S10	IF- 1:500
Src	Sc-19, Santa Cruz Biotechnology	Rabbit	8, S9	W- 1:1000

*IF- Indirect Immunofluorescence, W- Western Blotting, IP- Immunoprecipitation.

