# Supplementary material for: Regulation of E-cadherin localization by microtubule targeting agents: rapid promotion of cortical E-cadherin through p130Cas/Src inhibition by eribulin
Source: Oncotarget. 2017 Dec 31;9(5):5545–61. doi: 10.18632/oncotarget.23798 (PMC5814157; doi:10.18632/oncotarget.23798)
Supplement: Supplementary file 1 [file oncotarget-09-5545-s001.pdf]

Regulation of E-cadherin localization by microtubule targeting agents: rapid promotion of cortical E-cadherin through p130CAS/Src inhibition by eribulin

SUPPLEMENTARY MATERIALS

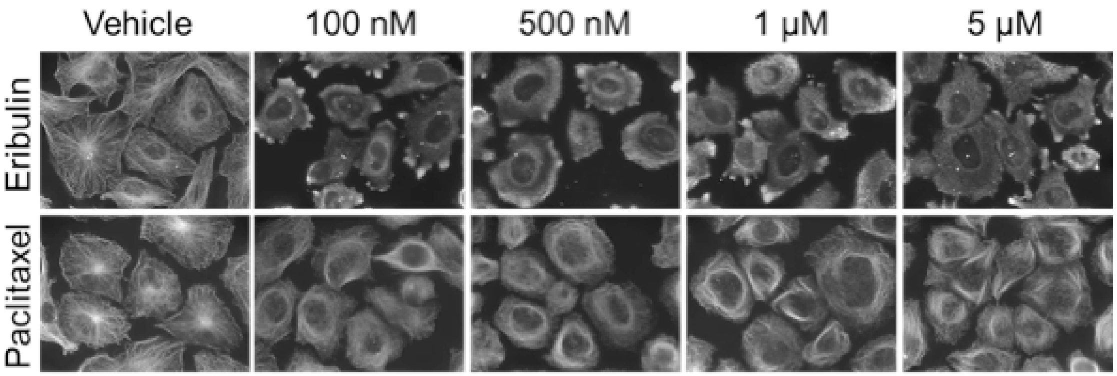

Supplementary Figure 1: Concentration-dependent effects of MTAs on interphase microtubules. BT-549 cells were treated for 2 hours with eribulin (top row) or paclitaxel (bottom row) at indicated concentrations. The interphase microtubule network was fixed and visualized by indirect immunofluorescence techniques using a β-tubulin antibody. Images are composed of non-deconvolved stacks.

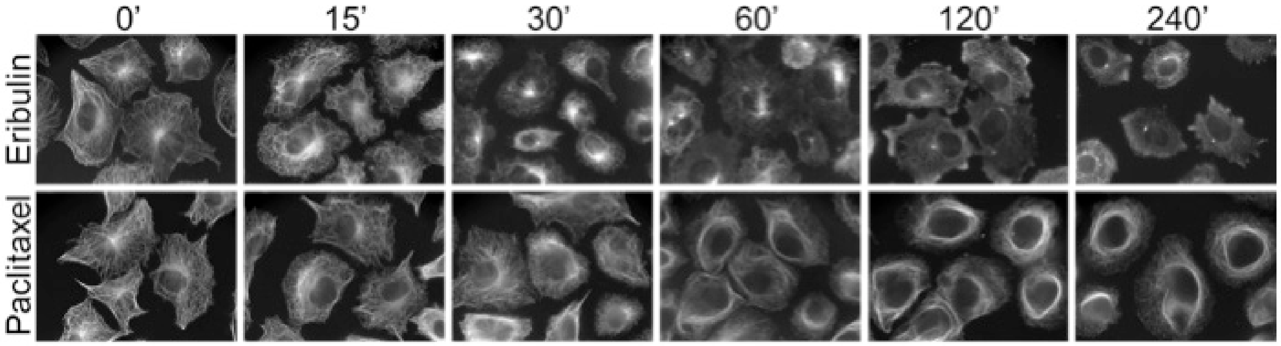

Supplementary Figure 2: Time-dependent effects of MTAs on interphase microtubules. BT-549 cells were treated with 100 nM eribulin (top row) or 1 μM paclitaxel (bottom row) for 0 – 240 minutes. The interphase microtubule network was fixed and visualized by indirect immunofluorescence techniques using a β-tubulin antibody. Images are composed of non-deconvolved stacks..

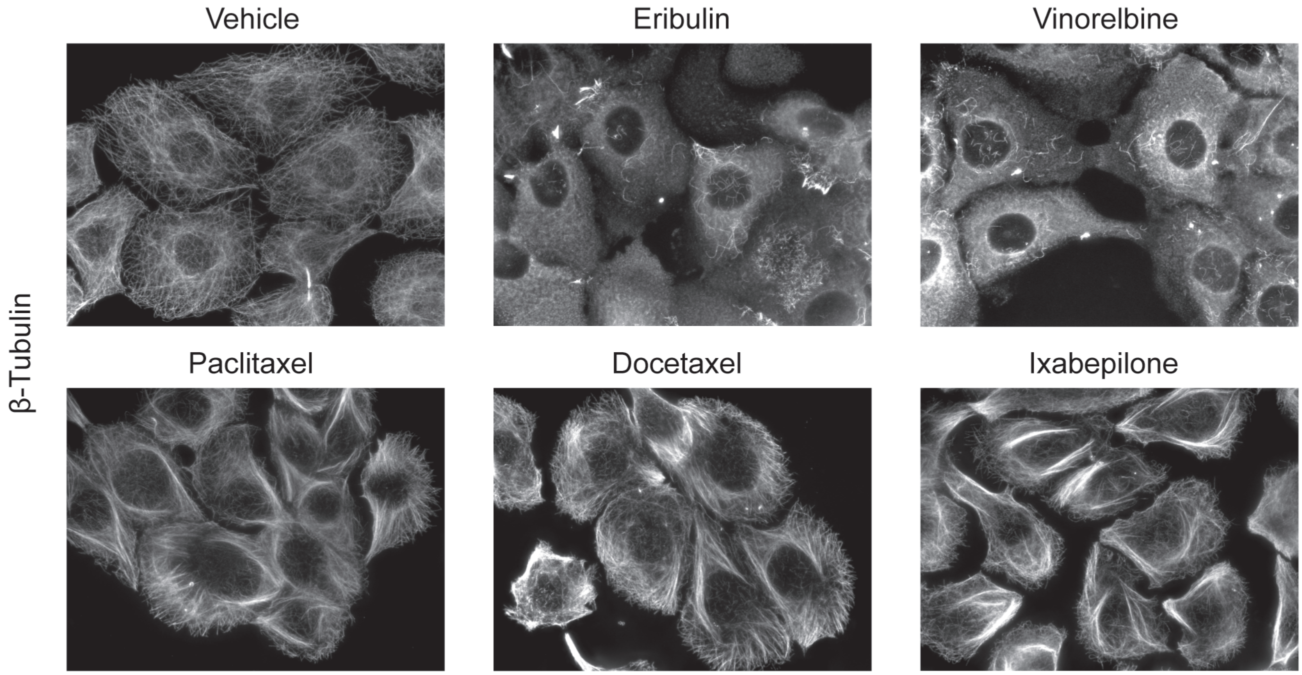

Supplementary Figure 3: Effects of a 2 hour incubation of MTAs on cellular microtubules. HCC1937 cells were treated for 2 hours with vehicle, 100 nM eribulin or vinorelbine or 1 μM paclitaxel, docetaxel or ixabepilone and microtubules visualized by indirect immunofluorescence. Images are composed of non-deconvolved stacks..

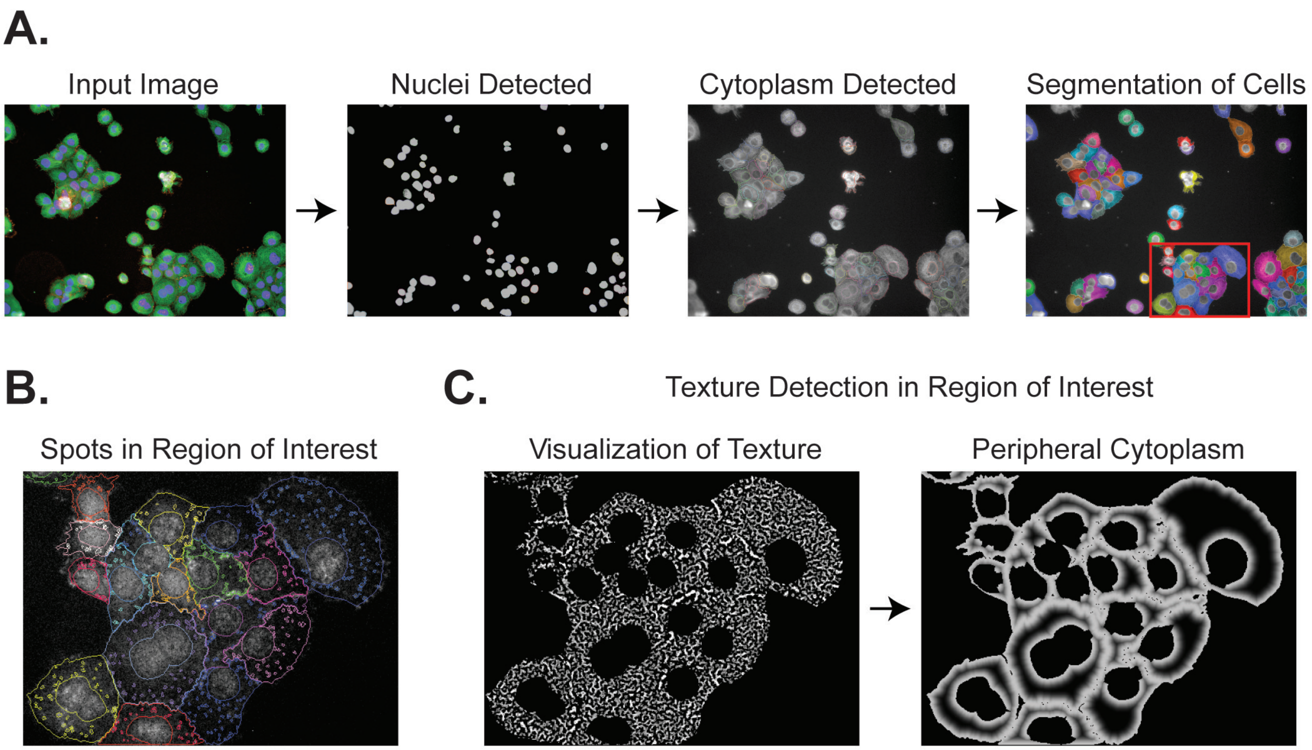

Supplementary Figure 4: High content image analysis of protein localization. **A**. Cells were imaged in a 96-well viewing plate using the Operetta® high content imaging system using a single plane of acquisition; untreated cells are shown in this figure as an example. A minimum of 9 different fields were evaluated for each treatment group. Columbus™ image analysis software was used to process and analyze the input images. Nuclei were detected using Method B in the Columbus software using a Hoechst stain. Surrounding cytoplasm was then detected using Method A in the Columbus software based on a β-tubulin stain or other cytoplasmic protein. MTAs did not affect the ability of the cytoplasm to be detected by this algorithm. Cells were then partitioned into cytoplasmic and nuclear segments that were used as regions of interest for two different methods of image analysis: spot and texture detection. **B**. The localization of proteins were quantified using spot detection Methods A and B in the Columbus software. This method detected spots in a defined region. The regions for these analyses were defined as the entire cytoplasm or the cytoplasm excluding the plasma membrane. There were several tunable parameters to determine what qualified as a spot which were kept consistent among all groups in a single analysis. **C**. The localization of proteins was also quantified using the STAR morphology classifications in the Columbus software. This analysis divides the cell into 5 regions (profiles) and then measurements of the symmetry and texture of a stain are quantified in those areas. A texture based analysis takes into account the spatial regularity of a stain, meaning the smoothness or roughness of the signal, which can give very different localization information than possible with spot detection alone. This texture analysis uses SER-features (Spots, Edges, and Ridges) which employ Gaussian filters to derive 8 features of a stain's distribution. The output measurement is the intensity (signal) of the resultant filtered image (an example shown in the left image). The SER-Ridge feature was used for all texture measurements as it nicely captures the membrane structures observed for both E-cadherin and P-Src (note the long white ridges seen at the periphery). The ridge measurements in the periphery of the cell (the outermost profile shown as the white peripheral ring in the right image) were used as membrane or cortical signal.

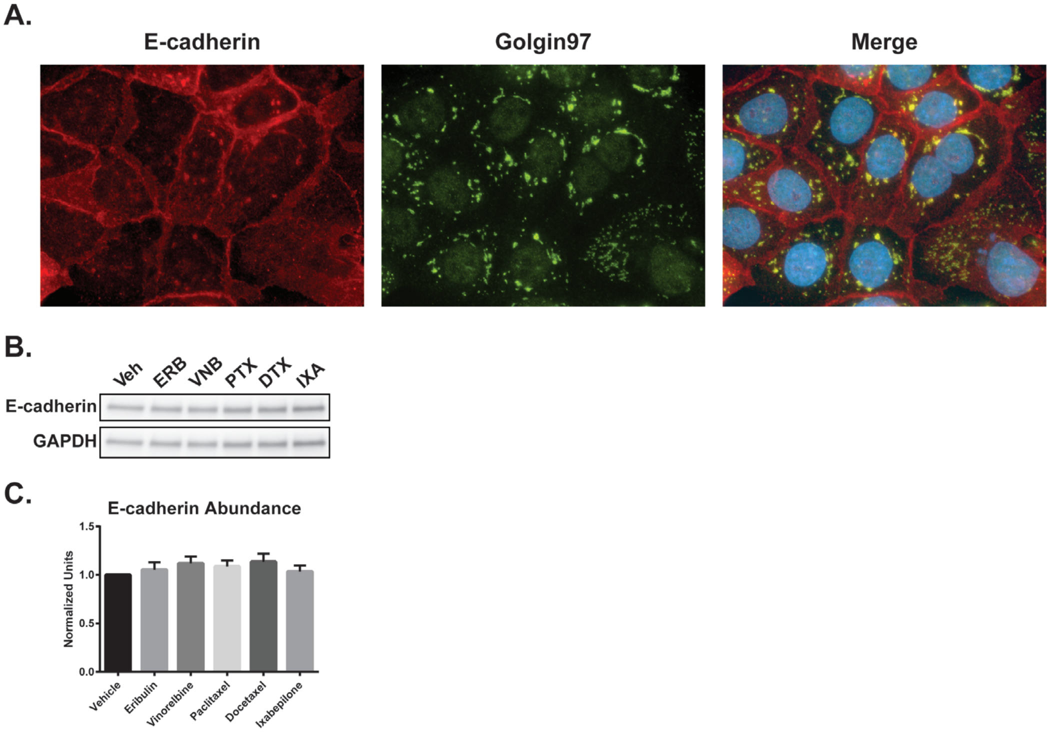

Supplementary Figure 5: The co-localization of the E-cadherin and golgin97 in paclitaxel-treated cells and effects of MTAs on E-cadherin abundance. **A**. HCC1937 cells were treated for 2 hours with 1 µM paclitaxel, cells fixed with paraformaldehyde and stained with E-cadherin and golgin97, and localization evaluated by indirect immunofluorescence. Images are composed of non-deconvolved stacks. **B**. HCC1937 cells were treated for 2 hours with 100 nM eribulin or vinorelbine or 1 µM paclitaxel, docetaxel or ixabepilone and E-cadherin was evaluated by immunoblotting of whole cell lysates. **C**. The relative protein levels of E-cadherin were quantified from multiple experiments. N = 4 ± SEM.

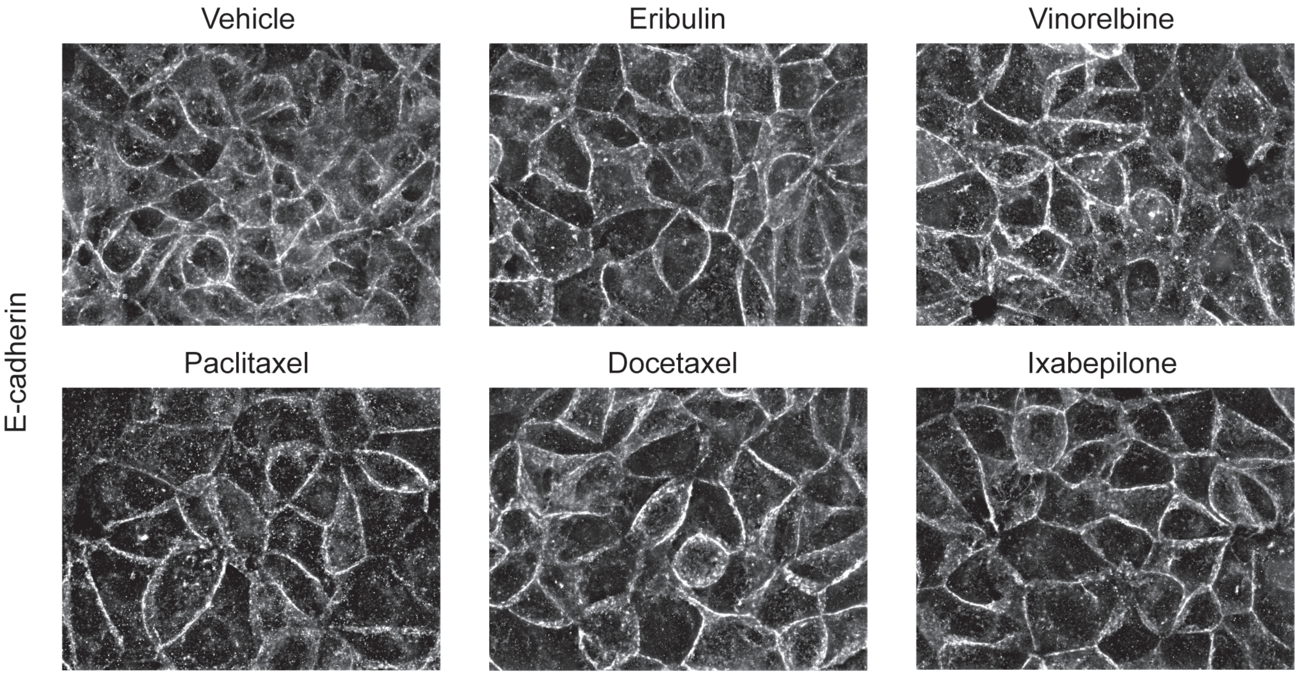

Supplementary Figure 6: Effects on E-cadherin localization after a 2 hour incubation in MCF-7 cells with vehicle, 100 nM eribulin or vinorelbine or 1 µM paclitaxel, docetaxel or ixabepilone as visualized by indirect immunofluorescence. Images are composed of non-deconvolved stacks.

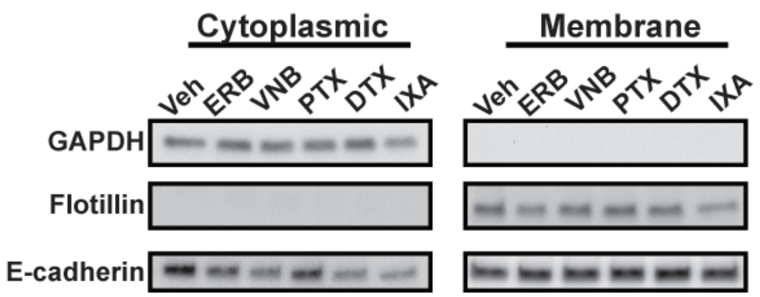

Supplementary Figure 7: Representative immunoblot of membrane and cytoplasmic-enriched lysates of HCC1937 cells treated for 2 hours with vehicle or MTAs.

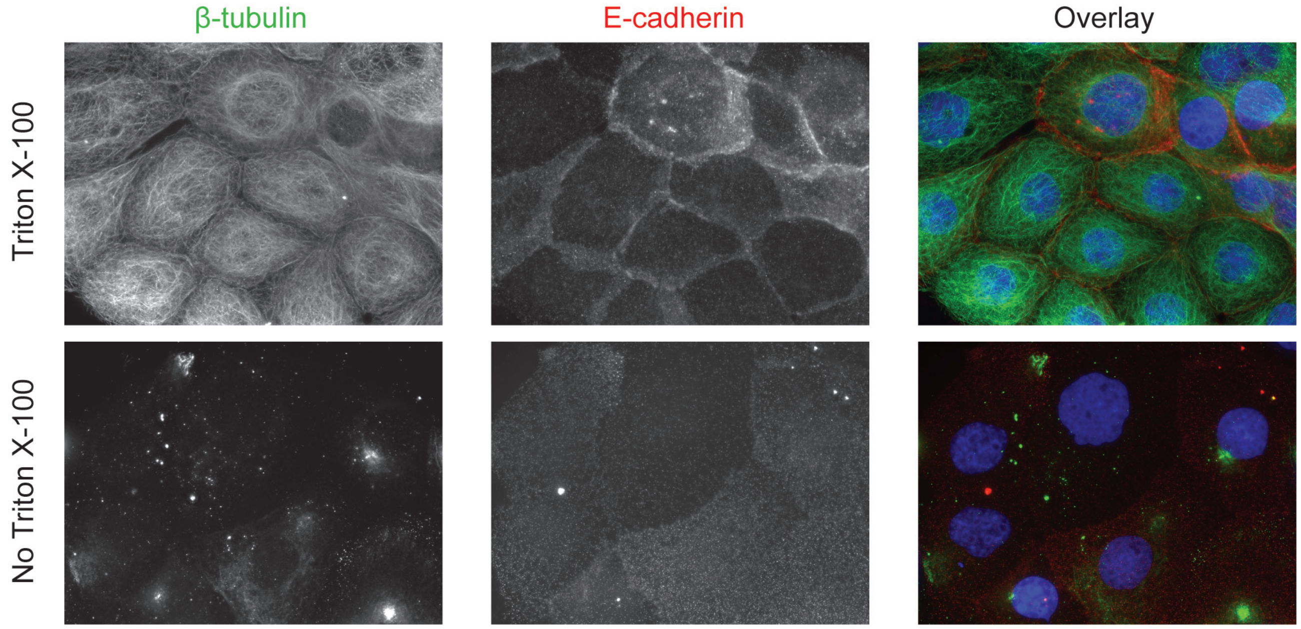

Supplementary Figure 8: Comparison of microtubule and E-cadherin staining between permeabilized and non-permeabilized HCC1937 cells. Cells were treated with vehicle and then prepared for indirect immunofluorescence with or without Triton X-100. Cells were probed for β-tubulin (green), E-cadherin (red) and Hoechst-33342 (blue). Images are composed of non-deconvolved stacks.

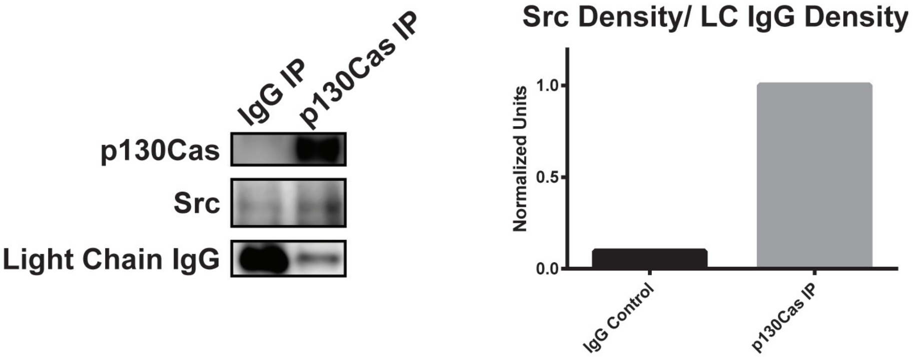

Supplementary Figure 9: IgG pull down control for Src co-immunoprecipitation with p130Cas. Immunoprecipitations of p130Cas or control IgG from HCC1937 cell lysates were subjected to western blotting for p130Cas, Src and light chain IgG. Quantification of Src densitometry normalized to the IgG densitometry is shown on the right.

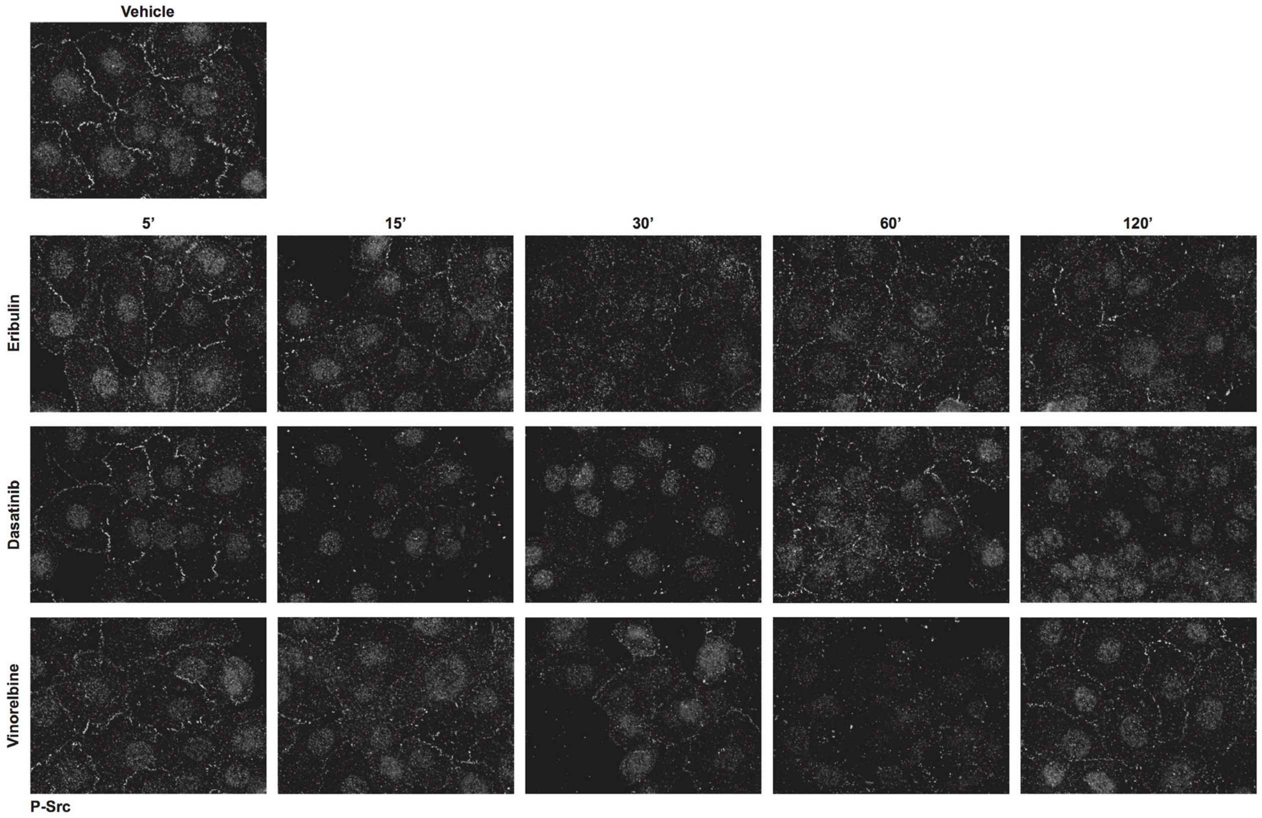

Supplementary Figure 10: Kinetics of dasatinib and microtubule destabilizers on P-Src localization. HCC1937 cells were treated with vehicle, 25 nM dasatinib or 100 nM of eribulin or vinorelbine for 5 -120 minutes. Deconvolved single channel image of P-Y418 Src corresponding to the merged image in Figure 9.

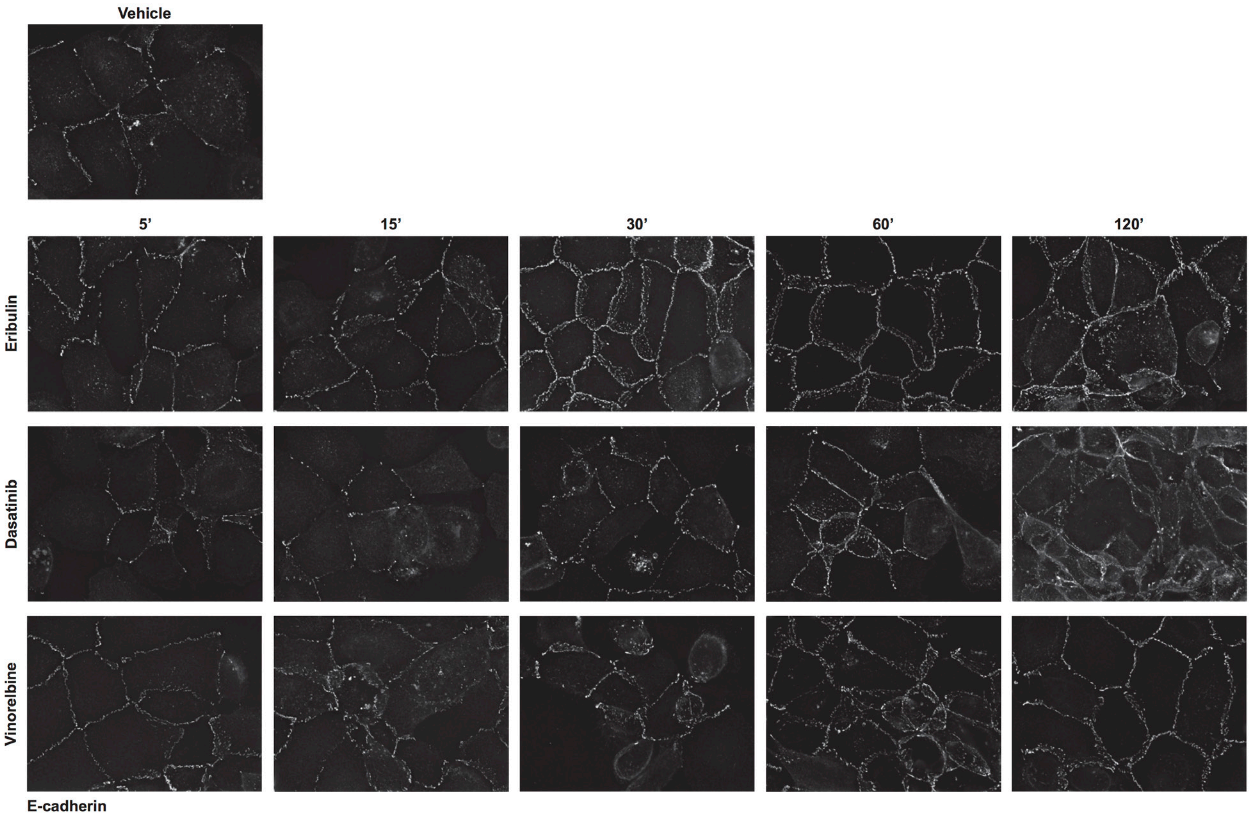

Supplementary Figure 11: Kinetics of dasatinib and microtubule destabilizers on E-cadherin localization. HCC1937 cells were treated with vehicle, 25 nM dasatinib or 100 nM of eribulin or vinorelbine over 5 – 120 minutes. Deconvolved single channel image of E-cadherin corresponding to the merged image in Figure 9.

Supplementary Table 1: Antibody Vendors and Applications

Host-Target	Vendor Information	Host	Figures Used	Application-Dilutions*
**E-cadherin**	3195S, Cell Signaling	Rabbit	3, 4, 5, 6, 7, S5, S6, S7, S8	IF- 1:400<br>W- 1:1000
**GAPDH**	5174S, Cell Signaling	Rabbit	6, S5, S7	W- 1:1000
**β-tubulin**	ab6046, Abcam	Rabbit	3	W- 1:1000
**β-tubulin**	T-4026, Sigma Aldrich	Mouse	1, 2, S1, S2, S3, S4, S8	IF- 1:400
**E-cadherin**	5296S, Cell Signaling	Mouse	9, 10, S11	IF- 1:400
**Flotillin**	BD Bioscience	Mouse	S7	W- 1:1000
**Golgin97**	A21270, Invitrogen	Mouse	S5	IF-1:200
**p130Cas**	ab31831, Abcam	Mouse	6, 8, S9	IF- 1:400<br>W- 1:1000<br>IP- 2 µg
**P-Y418 Src**	ab4816, Abcam	Rabbit	6, 7, 8, 9, 10, S4, S10	IF- 1:500
**Src**	Sc-19, Santa Cruz Biotechnology	Rabbit	8, S9	W- 1:1000

*IF- Indirect Immunofluorescence, W- Western Blotting, IP- Immunoprecipitation
